# Does fly larval cooperative behavior protect against parasitic wasps?

**DOI:** 10.1007/s00359-026-01799-0

**Published:** 2026-03-28

**Authors:** Kayli I. Morris, Barry Condron

**Affiliations:** Department of Biology, University of Virginia, Charlottesville, VA 22901 USA

**Keywords:** Parasitic wasps, Drosophila, Larval clustering, Behavior

## Abstract

Larval *Drosophila* exhibit cooperative foraging behavior in which groups of animals synchronize digging movements to form deep feeding cavities. Such clustering has been hypothesized to provide protection from predators and parasitoids by reducing surface exposure. Here, we tested whether clustering by *Drosophila melanogaster* larvae protects against parasitism by two *Leptopilina* species—*L. boulardi* (Lb), a specialist that primarily targets *D. melanogaster*, and *L. heterotoma* (Lh), a broadly infective generalist. Using controlled manipulations of larval number, sensory disruption, and environmental conditions, we examined how clustering affects wasp behavior, wasp mortality, infectivity, and the behavior of infected larvae. Contrary to expectation, larval clusters did not repel wasps, increase wasp mortality, or reduce parasitism. Instead, clustering increased the probability of successful infection, and Lb—but not Lh—infection altered host behavior in ways that promoted prolonged cluster residence. Lb infected larvae exhibited reduced coordination yet remained within clusters significantly longer than uninfected animals. Finally, the survival of infected larvae decreased in the presence of increasing numbers of uninfected siblings, an effect dependent on clustering. These results potentially reveal a bidirectional interaction in which parasitism enhances larval clustering and clustering enhances parasitism. Our findings challenge the assumption that cooperative digging evolved as an anti-parasitoid defense and instead suggest that parasitoid exploitation of social microenvironments may shape larval group behavior.

**Supplementary Information:**

The online version contains supplementary material available at 10.1007/s00359-026-01799-0.

## Introduction

Cooperative foraging behavior is widespread among dipteran larvae and is well characterized in *Drosophila melanogaster *(Dombrovski et al. 2017; Kuhar et al. 2024; Liao et al. 2024). When feeding on soft substrates, larvae form cohesive clusters that dig coordinated cavities, allowing them to access deeper food layers that might otherwise be unavailable to individuals. Cluster formation depends on sensory cues including vision and mechanosensation and requires fine-scale temporal coordination between neighbors (Dombrovski et al. 2019). The resulting social structure can influence larval fitness and development (Dombrovski et al. 2020; Williamson et al. 2021).

Social behavior is often seen as a potential protection against predators (Freeberg 2022). Indeed, the depth of digging in fruit is thought to protect *Drosophila* larvae against wasp predation (Carton and David 1985). By clustering, larvae are able to excavate deeper into the food substrate than individuals could alone (Dombrovski et al. 2017). This raises the question of whether clustering acts as a defense mechanism to protect larvae from wasps. Physical impediment of ovipositor entry due to minimized surface exposure in deep feeding cavities may reduce contact with predators or parasitoid wasps, and coordinated group movements may disrupt attacks. Hymenopteran parasitoids such as *Leptopilina boulardi* (Lb) and *Leptopilina heterotoma* (Lh) are major sources of larval mortality in natural *Drosophila* populations, using elongated ovipositors to inject eggs into second (L2) and third instar (L3) larvae (Wajnberg et al. 1990; Schlenke et al. 2007; Davis and Schlenke 2022). Because attacks are typically thought to occur near the food surface, a cluster-generated cavity might reduce the likelihood of stings or physically impede wasp entry.

Despite these assumptions, the behavioral interactions between parasitoids and larval clusters have not been examined. Furthermore, parasitic infection itself could alter larval social behavior, potentially generating feedback loops that shape both cluster dynamics and parasitism outcomes (Wajnberg et al. 1990; Schlenke et al. 2007; Davis and Schlenke 2022). Lb and Lh differ in host specificity, venom composition, and effects on host physiology, offering a comparative system for understanding how parasitism interacts with larval behavior.

By experimentally manipulating larval density, sensory cues, and wasp species, we show that clustering does not act as a defensive barrier but instead increases susceptibility to parasitism. Surprisingly, infection by Lb increases clustering by prolonging the residence time of poorly coordinated larvae. These results suggest a previously unrecognized interaction between parasitism and cooperative behavior in *Drosophila* larvae.

## Methods

### Fly stocks

**Table:** 

Designation	Description	Species	Notes
CS	CantonS	*D.melanogaster*	Ed Lewis, Caltech
GMRhid	Blind	*D.melanogaster*	Bloomington #5771

### Fly stock maintenance and egg collection

All Drosophila melanogaster strains were raised in standard Caltech food vials containing (1000 ml molasses, 14000 ml H_2_O, 148 g agar, 1000 ml corn meal, 412 g Baker’s Yeast, 225 ml Tegosept, 80 ml propionic acid). Pre-processed vials were prepared as described (Dombrovski et al. 2020). Video analysis was as described (Dombrovski et al. 2017). Larvae were kept at 24 °C, 30% humidity. For egg production, ~ 50 adult flies 3–4 days old were transferred into egg cups and kept in the same conditions. Eggs were collected on 35 mm petri dishes containing standard agar-molasses food and yeast.

### Wasp husbandry

Wasps (Lb17, Lh14) were obtained from Dr. Dan Tracey and Dr. Lydia Borjon at U. of Indiana. These were kept in 25 mm vials with about 100 CS larvae. Flug-style stoppers were dipped, wasp side, in Karo-syrup as food for adult wasps. Wasps were passaged between vials about every 3 weeks. For sorting, wasps were knockout with CO2 in the same manner as *Drosophila* adults and males identified by their long antennae.

### 2D cluster assays

Cluster assays were performed as described except that an artificial medium of 1%agar, 2% starch and 2% yeast extract was melted and allowed to cool and set between the glass slides. Video documentation was either performed and analyzed with an iPhone 4 or a Nikon digital SLR with a macro lens, as described (Liao et al. 2024).

### Photography and Video recordings

For cluster frequency analysis, videos were recorded on an iPhone 4 at full resolution and 1 frame/60″ using “Lapseit” software for iOS. For wing images, an iPhone 10 was used. Video analysis was further performed in iMovie and ImageJ (32-bit version for Windows). For synchronization measures, a Nikon D3100 CMOS camera, with 50mm lens and fitted with a Raynox Macroscopic 4 × lens was used and videos (1920 × 1080 pixels) were recorded at 24Hz. Synchronization was measured between pairs of larvae in a cluster as described (Kuhar et al. 2024; Liao et al. 2024).

### Statistical analysis

Unless otherwise stated, all data are presented as mean values and error bars represent standard deviation. Statistical significance was calculated by one-way ANOVA using Tukey’s method. For most measures, the standard control standard is 40 CS larvae in a vial or 2D cluster apparatus. When comparing two groups of normally distributed data, Student’s two-tailed unpaired *T*-test was used. *p < 0.05; **p < 0.01; ***p < 0.001. Analysis was conducted using the GraphPad Prism 8 statistical software.

## Results

Many dipteran larvae form social foraging clusters which can dig deep into food and might, in part, be a way to escape parasitic wasps. Clustering larvae generate a tunnel into the food substrate (Dombrovski et al. 2017). Clusters might protect against wasps in two ways: by drowning predators trapped in deep cavities, or by preventing wasps from entering these structures. These hypotheses were tested in the following experiments. While most fly larvae cluster, in this paper we focus on *Drosophila melanogaster* CantonS (Dmel) prey as it is the most robustly tested larvae population for clustering parameters. Two parasitic wasps were used, *Leptopilina boulardi* (Lb) and *Leptopilina heterotoma* (Lh), which both prey on CantonS (CS) larvae but have differing features—Lb using a passive immune evasion that prevents immediate immune cell death in the host, while Lh injects an active immune suppression venom that directly kills host immune cells (Schlenke et al. 2007). However, both wasps inject a complex cocktail of immune- and neuro- affecting factors that alter many aspects of host function (Schlenke et al. 2007).

### Observations of wasps around clusters

**Fig. 1:**
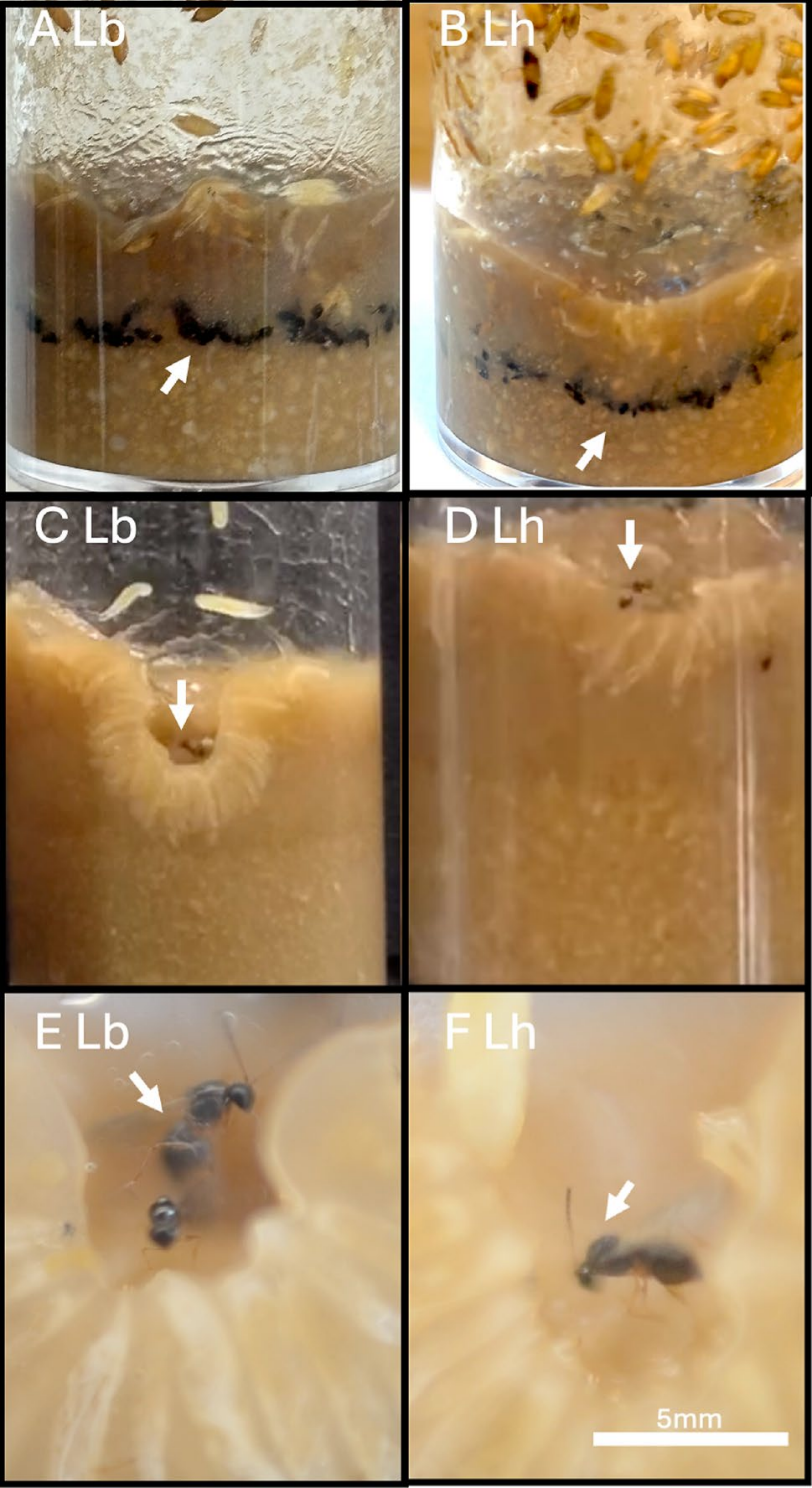
Observations of wasp behavior. **A**–**B**. Lb (**A**) and Lh (**B**) wasp death is seen as a layer of carcasses (arrow). **C**–**D**. Magnified view of clusters in vials showing Lb (**C**) and Lh (**D**) wasps in clusters. E. Hi-resolution still images from videos of wasps in clusters, Lb (**E**) and Lh (**F**)

CantonS larval clusters were established as described (Liao et al. 2024). About 100 L2 larvae were added to a pre-used 25 mm vial and allowed to form clusters after 3 days. About 50 wasps were added, a mixture of male and female adults. Wasp behavior was video recorded from the side and from above at 6 frames/minute. Wasps of both species are seen to regularly enter clusters (Fig. 1). In addition, while many dead wasps were seen buried in the food, no cases of these getting caught specifically by clusters were documented. High-resolution video recording was also conducted and, aside from some retreat movements to the ovipositor, larvae and clusters remained unperturbed by wasp presence (Movie S1a–d). The conclusion from these observations is that while wasp death does occur with larvae, it was not observed specifically in clusters and that wasps enter these structures quite freely. Therefore, wasp death was measured specifically in the context of differing amounts of clustering.

### Do clusters specifically kill wasps?

**Fig. 2:**
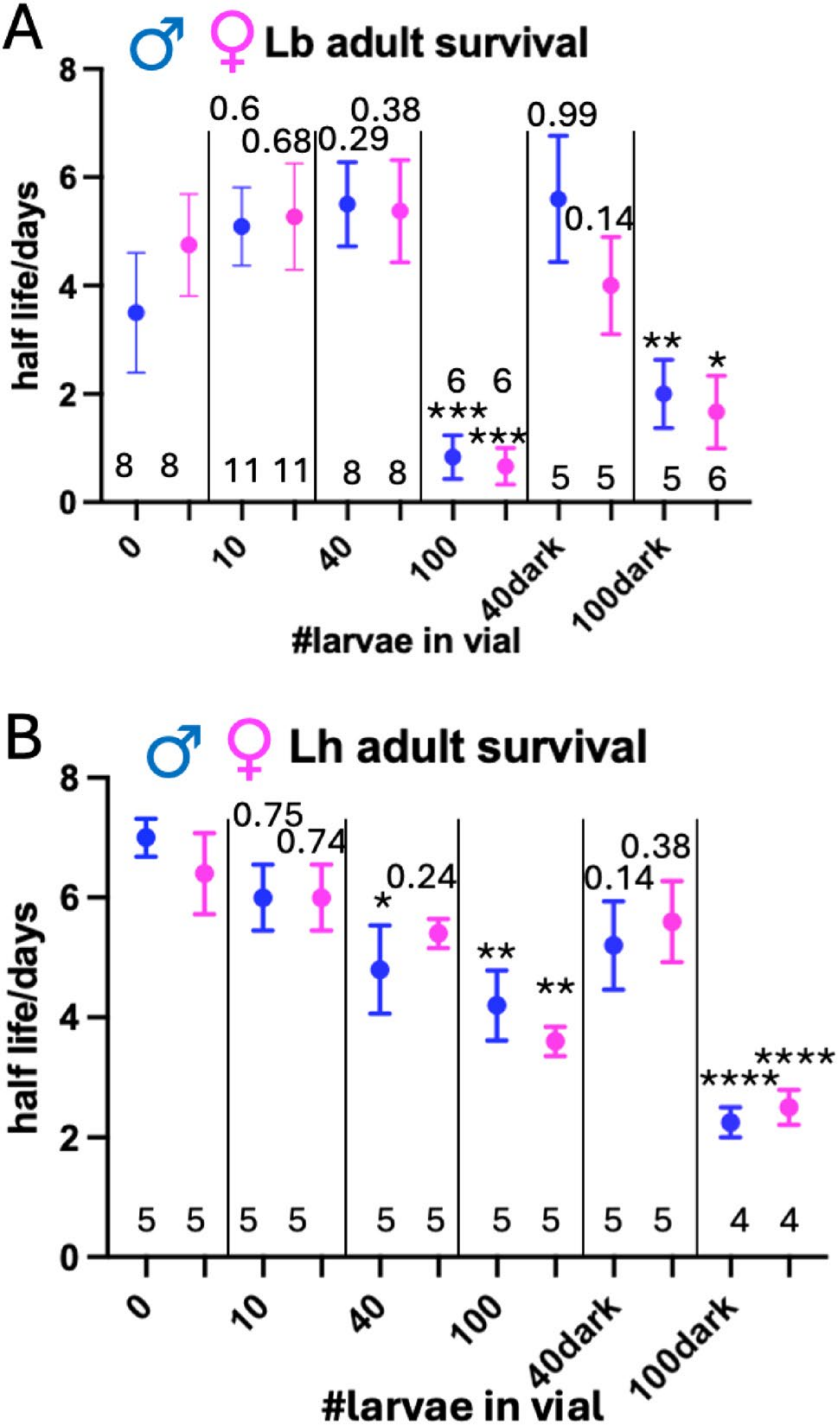
Adult wasp death. **A** Number of days for over 2 of 5 added male (blue) or 5 female (pink) Lb wasps to end up drowning in food. Number of vials used for each measure indicated. Statistics were compared using ANOVA and significance or probabilities are indicated when non-significant. **B** Same as 2**A**. except Lh wasps

To measure wasp death, 5 male and 5 female wasps were placed in a vial with 100 L2 larvae which begin clustering the next day (Fig. 2). The rationale for using male and female is that females might be more susceptible to drowning as they spend more time on the food stinging. At least 4 vials were used for each measure and wasps counted every day. The number of days needed to go from 3 to 2 surviving wasps of each gender was determined and plotted (Fig. 2). Varied larval conditions were used: 0 and 10 larvae will have no clustering while 40 and 100 larvae do cluster (Liao et al. 2024). Placing 40 or 100 larvae in the dark greatly reduces clustering as it has been previously shown that vision is required for clustering (Dombrovski et al. 2019). Larvae with genetically induced blindness is observed to enter clusters and interfere with the required coordination, thus dismantling the structures (Dombrovski et al. 2019, 2020). No male/female differences were seen across both species and in all clustering conditions. This indicates that stinging behavior specifically does not potentiate drowning. Both Lb and Lh wasps show increased mortality with increasing numbers of larvae, but this is not offset by maintaining vials in the dark. Therefore, while larvae in high numbers do kill wasps, this is not likely based on clustering behavior.

### Does clustering affect wasp infectivity?

**Fig. 3:**
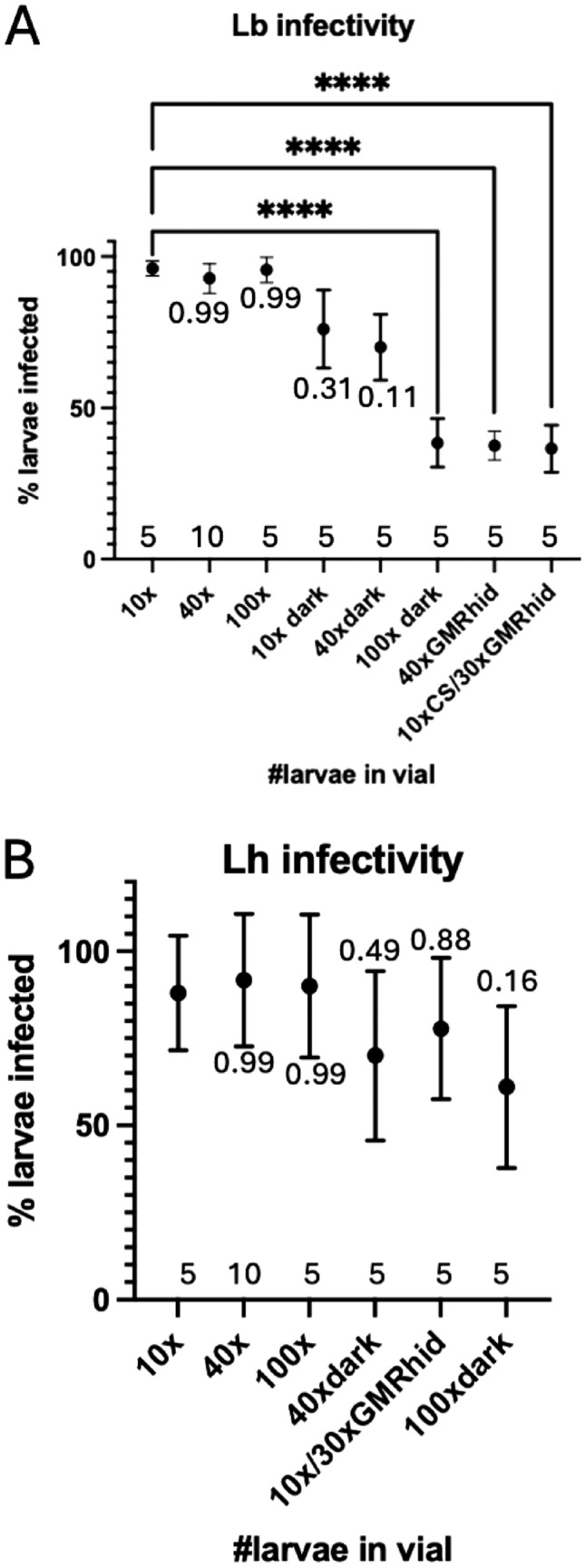
Wasp infectivity. **A** Infectivity was calculated as percentage larvae successfully infected with wasp eggs. Statistics were compared using ANOVA and significance or probabilities are indicated when non-significant. **B** Same as 3**B** except using Lh wasps

While larval clusters do not seem to potentiate the capture and drowning of wasps, they could reduce infectivity by making it more difficult to sting. To examine infectivity, 10 wasps (5 male and 5 female of both species) were placed in vials along with various numbers of larvae. Upon complete pupation, all wasps were removed and incubation completed to measure the number of wasps. Infectivity was calculated as the number of wasps produced per larva in each vial. For Lb, infectivity was about 90% for vials of clustering larvae independent of population size above 40 (Fig. 3a) which is similar to previous reports (Jones and Hurst 2020). However, upon reduction of clustering either via incubation in the dark or addition of clustering-spoiler blind larvae, infectivity is reduced significantly. These data indicate that clustering, in fact, favors infectivity. In contrast to Lb however, Lh shows a steady 80% infectivity across all larval states. These data indicate clustering enhances infection success for Lb but not for Lh, revealing a species-specific interaction between parasitoid strategy and larval social behavior. This raises the question, if clustering favors infectivity, how might parasitic infection affect larval clustering?

### How does infection affect clustering?

**Fig. 4:**
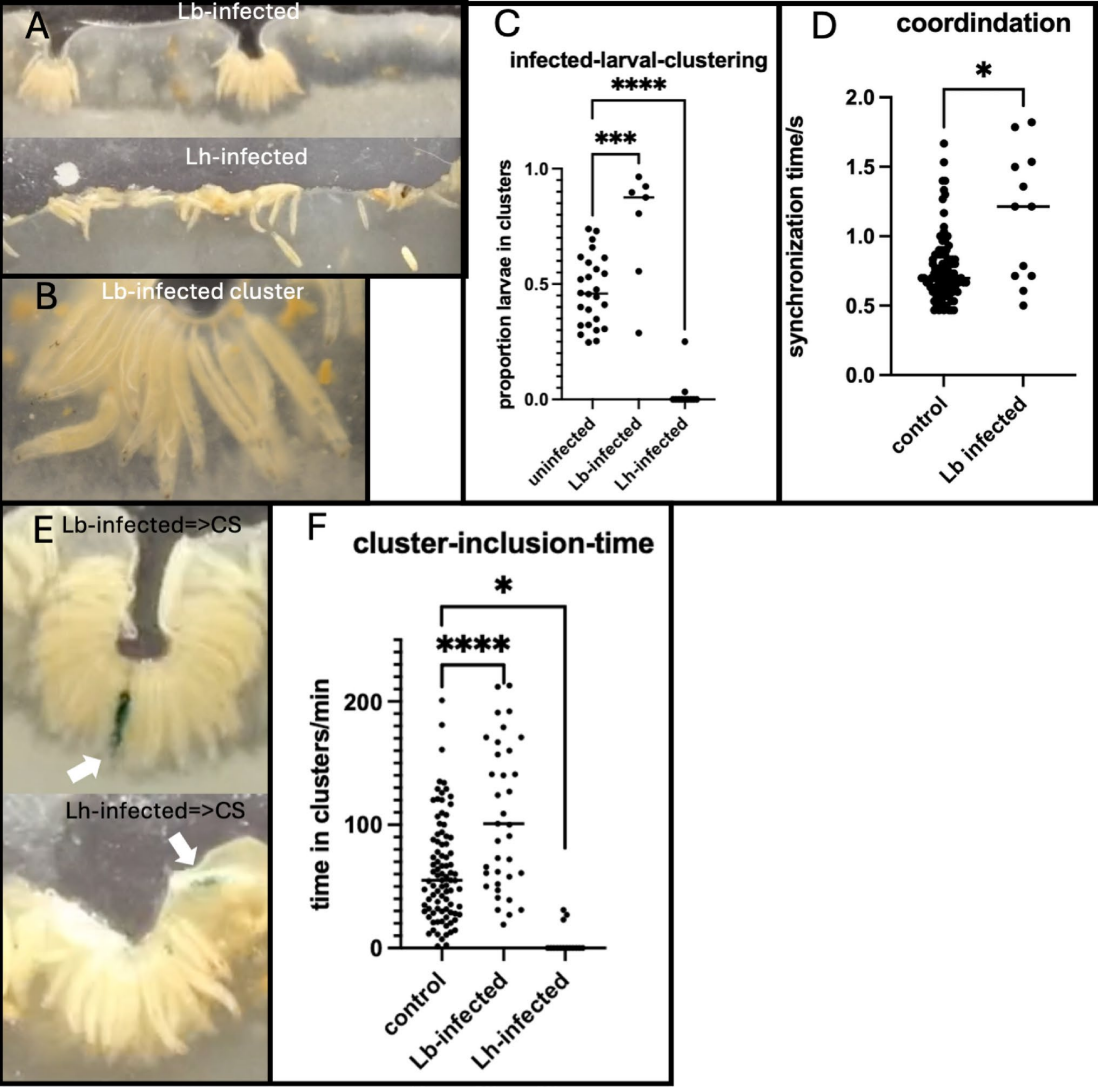
Infected larval clustering. **A** Larvae infected with Lb (upper panel) or Lh (lower panel) were placed in a 2D cluster assay. Robust clusters form for Lb infection but not for Lh. **B** Close up of a Lb-infected larval cluster. Larval posteriors are not as well aligned as generally observed. **C** Clustering is higher in Lb-infected than for control. Only one brief cluster was seen for 11 Lh infected larval assays. Control is from a previous study(Dombrovski et al. 2017). **D** Infected larval (arrow) inclusion into an uninfected cluster. **E** Coordination of larvae was calculated from high resolution videos. **F** Cluster inclusion time for Lb and Lh infected larvae. 15 Lh-infected larvae were examined and only 3 entered clusters briefly. Control is from a previous study (Dombrovski et al. 2017). Statistics were compared using ANOVA and significance or probabilities are indicated

To measure the effect of infection on clustering, infected larvae were generated by placing 40 L2’s in the vial with at least 20 wasps of both species. Larvae were removed 4 days later, at the third day of the L3 stage, at which time maximal clustering is most often achieved. Under these conditions, over 90% of larvae are infected (Fig. 3). Infected larvae were placed in a 2D clustering assay and monitored for behavioral changes. Clusters were observed for Lb-infected larvae but not for those of Lh (Fig. 4a). Lb clusters, however, appeared more disorganized than those of uninfected CS (Fig. 4b). Specifically, larvae posterior spiracles are not as aligned as is generally observed (Dombrovski et al. 2017). Clustering was measured as described by averaging the number of larvae in clusters over 3 time points per assay. Compared to uninfected CS, Lb-infected larvae cluster more and Lh do not. Because inclusion in clusters requires coordination between larvae, it is possible that Lb-infected larvae might be better at this synchronization. However, measuring the coordination between larvae using high-resolution video recording (Fig. 4d) indicates the opposite. Uninfected larvae coordinate with a 0.6″ delay: when one moves, its closest neighbor moves about a half second later. Infected larvae delay on average 1.2″, which is nearly statistically random for a 2″ locomotion cycle, or no coordination (Dombrovski et al. 2017). Therefore, some means, other than coordination, keeps these larvae in clusters, which has been observed for salivary-gland-depleted larvae (Liao et al. 2024). To test whether Lb-infected larvae stay in uninfected clusters longer, food-coloring-labeled infected animals were added to uninfected clusters and the time residing in the cluster measured (Dombrovski et al. 2017; Liao et al. 2024). Lb-infected larvae were observed to spend about twice as long in a cluster over uninfected (Fig. 4f). Lh-infected larvae do not enter clusters (Fig. 4e). In conclusion, Lb infection appears to increase clustering by increasing the average time a larva resides in a cluster.

### Do uninfected larvae affect the survival of the infected?

**Fig. 5:**
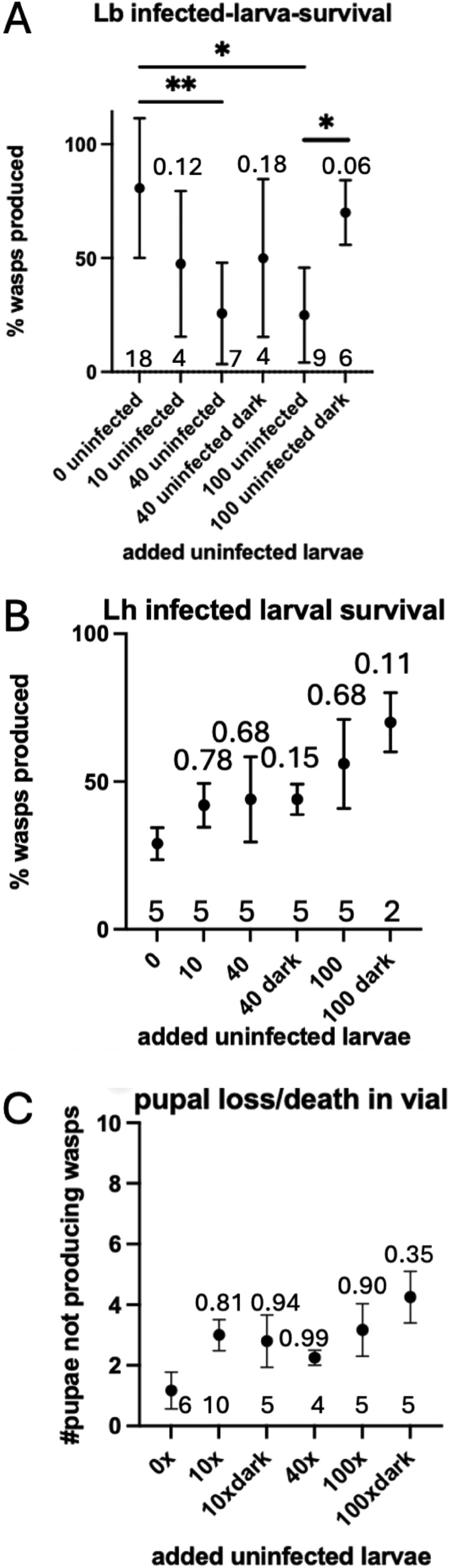
Survival of infected larvae. **A** 10 Lb infected early third instar larvae were added to a vial and survival measured as final wasps produced. Uninfected age-matched sibs were added at indicated numbers. **B** Same as 5A except that Lh infected larvae were used. **C** Lb infected pupal death was measured by allowing 10 larvae to pupate and then monitor them until wasp hatching. Various number of uninfected larvae were added which pupated around their infected sib. Pupae that were lost off the side of the vial were scored as lost. For **A**, **B** and **C**, the number of vials used for each measure indicated. Statistics were compared using ANOVA and significance or probabilities are indicated when non-significant

Previous studies have indicated that stung larvae can be subjected to cannibalism from those uninfected. Such behavior may confound the below measures. In the transplant experiments shown in (Fig. 4e), no Lb-infected larvae were observed being attacked by their uninfected neighbors. Lh larvae are regularly seen to sink (Movie_S4) below clusters, and this might be just due to lack of locomotor ability. To measure infected larval mortality, 10 early third-instar Lb- or Lh-infected larvae were placed in a vial with various numbers of uninfected cohorts. Mortality was measured by counting how many wasps came out of each vial. Lb-infected larvae show increased loss when there are increasing numbers of uninfected cohort-mates. In addition, incubation in the dark, which reduces clustering behavior (Dombrovski et al. 2017, 2019, 2020), decreases the Lb death rate as measured for 100 × uninfected larvae. Lh-infected larvae (Fig. 5b) have about a 50% survival rate uninfluenced by other uninfected cohorts residing within their vial. While no Lb-infected larvae were seen to be specifically attacked in clusters, it is possible that they are predated as pupae. To test this, 10 Lb-infected larvae were placed in a vial and allowed to pupate. These were marked in the vial. Various numbers of uninfected cohorts were added, and the pupae were counted every day for 5 days. Pupae that were removed and pulled into the food by uninfected larvae were counted. On average, about 2 out of 10 are lost off the vial side, regardless of the conditions (Fig. 5c). This does not account for the high mortality of Lb-infected larvae when with a high number of clustering cohorts.

### Correlations of findings with clustering

**Fig. 6:**
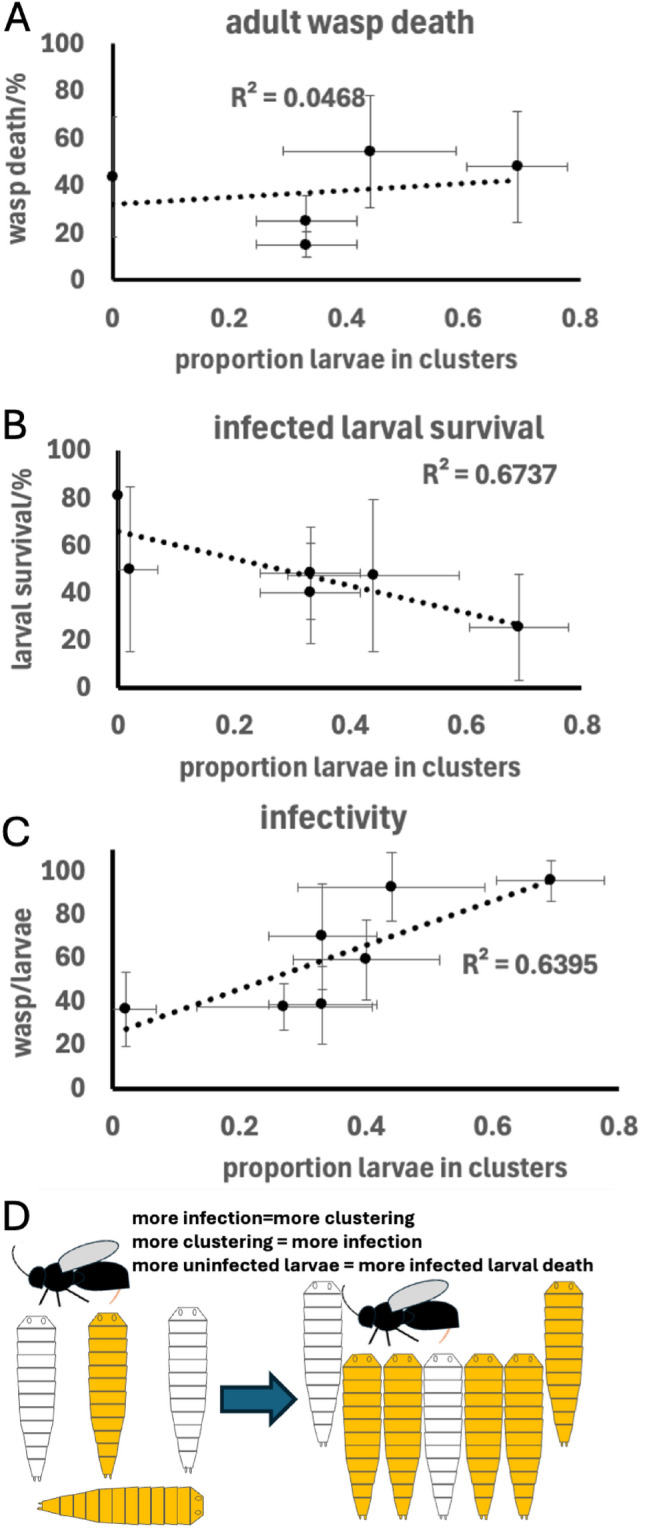
Summary of correlations with clustering. **A**–**C**. Clustering frequencies measured in previous studies(Dombrovski et al. 2017, 2019, 2020; Williamson et al. 2021; Liao et al. 2024) were compared to adult wasp death (**A**), infected larval survival (**B**) and infectivity (**C**). The correlations were scored by R^2^ calculation. While adult wasp death is not related to clustering, infected larval survival and infectivity is. **D**. Model for the role of clustering in the parasitic wasp life cycle. Two phases might exist for Lb infected larvae: at low frequency these infected larvae are at high risk for being killed by the uninfected. At higher frequency of infection, larvae are drawn more into clusters which further increase the degree of infection

To summarize the findings in Figs. 1, 2, 3, 4, 5, cluster frequency was estimated from previous studies and compared to adult wasp death, infected larval death, and infectivity (Fig. 6). There is little correlation between the amount of clustering and wasp death (Fig. 6a). While wasps die more with more larvae, this is more likely a result of increased food fluidity than actual clusters. However, there is a correlation between clustering and infected larval death when there are uninfected cohorts. Countering previous hypotheses, this may not be due to cannibalism, as no such behavior was observed in any of the cluster transplants in 2D assays. However, these observational studies did not look outside of vial clusters for cannibalism or other signs of larval death from the many other changes in altered food. This might instead be due to infection through the sting site. Finally, there is a correlation between infectivity and clustering (Fig. 6c). The more larvae cluster, the more wasps can infect them.

## Discussion

Wasp parasitism is a major source of mortality in dipteran larvae and a powerful potential natural means of containing invasive populations such as that of *D. suzukii *(Fleury et al. 2004; Schlenke et al. 2007; Neupane et al. 2024). Many dipteran larvae form cooperative foraging groups, or clusters, and should such social behavior be a mechanism to avoid wasp stinging, studying it may allude to potential method of controlling invasive *Drosophila* populations (Neupane et al. 2024). Clustering results in deeper digging in food and larvae further from the surface are hypothesized to be less vulnerable to parasitic oviposition (Carton and David 1985). The objective of this study was to alter clustering specifically and measure the effects on wasp infection. Interestingly, the opposite effect was found: Clustering behavior increases stinging success, and infection in turn prolongs clustering structures. It can be speculated that wasps may in fact seek out clusters to find more larvae to infect, and after infection, more clustering may ensue.

Wasp death is extensive in fly vials, and carcasses are pushed deep into the food presumably by the action of clusters (Fig. 1a, b). In addition, larvae were observed to consume wasp carcasses (Fig Movie_S2). However, while it might seem attractive to view clusters as potential wasp traps, this is not the case. Wasps enter cluster cavities freely and engage in stinging behavior. Wasp death is likely caused by the liquidity of the inhabited food, which is dependent upon larval numbers and secretions. Therefore, clustering does not kill parasitic wasps. While social behavior in animals is often explained in terms of escape from predators, in the case of parasitic wasps in the vials used in these experiments, this is not observed. Some larvae, like *Megaselia*, dig more deeply into the food, and drier food conditions might also allow longer tunnels such that there might be conditions in which clustering does protect against wasps (Kuhar et al. 2024).

In discussing the hypothesis that uninfected larvae appear to kill those infected, it seems this behavior is dependent upon clustering activity. While no cases were observed where uninfected neighbors attacked a single infected larva, it could nevertheless still be occurring via unexpected means. For instance, it might be unhealthy for larvae to spend too long in the cluster, and infected larvae are observed to spend twice as long as their uninfected counterparts. Reduced health via prolonged engagement in clusters may be due to waste buildup or lack of oxygen availability. Clustering dynamics might be optimized for food processing with regular breaks to regenerate a metabolic state. Whatever mechanism clusters may lead to infected larval death, it decidedly requires the presence of uninfected cohorts. There are some differences between stung and uninfected larvae—such as the sting site, aspects of their metabolism, or over-clustering behavior that makes them susceptible to uninfected (Fatouros et al. 2020; Kakeya and Takahashi 2021). Another possible explanation might reside in differences in microbiome: Wasp larvae establish their own microbiome which might change that of the host to weaken their health compared to those uninfected (Zhou et al. 2022).

A significant limitation of these studies is that the behavioral act of clustering likely results in many downstream confounding effects such as external food processing, nutrition as well as well as the external microbiome. While inclusion of blind larvae to disrupt clustering keeps parameters such as larval density the same, there are many potential downstream changes still to investigate. Clustering itself, while well studied, is still not understood in terms of food processing and this needs to further investigated to understand the effects of this behavior on wasp activity.

While clustering increases infectivity, this may be simply due to physical access of surface-concentrated larval posteriors. A wasp jabbing a stinger will have much more opportunity for a successful injection in a cluster than in a sparser area of a vial. In addition, evasive maneuvers such as rolling were not observed in clusters and might make larvae more susceptible. In fact, larvae in clusters tend to back up into the stinging wasp potentially making it easier to be infected (Movie_S1a). It is observed that Lh still infect larvae well in the dark. The increased Lh wasp infectivity in dark might be due to increased larvae on the food surface, potentially due to some level of reduced photophobia.

Finally, crawling on the exposed posteriors of larvae in a cluster might be a safer place for wasps to be in the liquid food and therefore a place they gravitate towards.

On the topic of infected larvae clustering more, this was observed in salivary-gland-depleted larvae and therefore may be a change in internal state (Liao et al. 2024). One explanation suggests that infected larvae may exhibit increased feeding drive. For instance, *Cordyceps* infection in caterpillars breaks down internal trehalose which is thought to make the host eat more (Zhao et al. 2025). Lb and Lh wasps may similarly modify host physiology to induce feeding behavior in order to improve conditions for parasitoid growth. It is also possible that Lb-infected larvae have specific locomotor deficits which makes it more difficult to leave clusters.

These studies raise the possibility that there are two phases of larval behavioral states presented to an attacking wasp. If wasps can enter a cluster and sting as many as possible, the clusters will last longer, attract more uninfected cohort-mates, and further increase the parasitism. Should this be the case, it would suggest wasps attempt to infect as many larvae as possible in a short time in order to initiate a positive feedback loop that increases clustering structure maintenance. However, if only a few larvae are infected, then the cluster-killing of infected larvae would decrease the success of parasitism. In both scenarios, wasps should attempt to flock to cluster cavities and sting as many larvae as possible. Should not enough successful stinging events occur, the infected hosts will be killed by the uninfected and the wasp will be unsuccessful in reproducing. Clustering dynamics may have evolved to limit cluster duration, thereby reducing the size and stability of the stinging target. Under such conditions, wasps may benefit from cooperatively locating and rapidly stinging clusters. The absence of clustering in *D. suzukii*—the only larval Drosophila species known to lack this behavior—may represent an adaptation that reduces parasitoid vulnerability (Kuhar et al. 2024). This presents important implications for the success of parasitoid-driven biocontrol approaches. In summary, clustering is not likely a simple way to avoid wasps. In some substrates, like those of the lab, clustering might be a vulnerability to wasp infection.

## Supplementary Information

Below is the link to the electronic supplementary material.

Supplementary file1 (MP4 4249 KB) Video of Lb wasps on cluster in vial

Supplementary file2 (MP4 988308 KB) Video from above of Lb wasps and of clusters which can be seen as cavities in the substrate

Supplementary file3 (MP4 32895 KB) Video of Lb wasp in a 2D culture

Supplementary file4 (MP4 1465437 KB) Video of wasp carcass being consumed by larvae

Supplementary file5 (MP4 176347 KB) 2D cluster with Lb infected larvae

Supplementary file6 (MP4 20442 KB) Blue food coloring-labeled Lb-infected larvae joining uninfected clusters

Supplementary file7 (MP4 6286 KB) Blue food coloring-labeled Lh-infected larvae around uninfected clusters

Supplementary file8 (MP4 762 KB) Blue food coloring-labeled Lh-infected larvae being pushed into medium by uninfected clusters

## Data Availability

No datasets were generated or analysed during the current study.
